# Intestinal Barrier, Claudins and Mycotoxins

**DOI:** 10.3390/toxins13110758

**Published:** 2021-10-26

**Authors:** Marta Justyna Kozieł, Maksymilian Ziaja, Agnieszka Wanda Piastowska-Ciesielska

**Affiliations:** Department of Cell Culture and Genomic Analysis, Medical University of Lodz, Zeligowskiego 7/9, 90-752 Lodz, Poland; marta.koziel@umed.lodz.pl (M.J.K.); ziaja.ziaja0@gmail.com (M.Z.)

**Keywords:** claudins, mycotoxins, intestinal barrier, tight junctions

## Abstract

The intestinal barrier is the main barrier against all of the substances that enter the body. Proper functioning of this barrier guarantees maintained balance in the organism. Mycotoxins are toxic, secondary fungi metabolites, that have a negative impact both on human and animal health. It was postulated that various mycotoxins may affect homeostasis by disturbing the intestinal barrier. Claudins are proteins that are involved in creating tight junctions between epithelial cells. A growing body of evidence underlines their role in molecular response to mycotoxin-induced cytotoxicity. This review summarizes the information connected with claudins, their association with an intestinal barrier, physiological conditions in general, and with gastrointestinal cancers. Moreover, this review also includes information about the changes in claudin expression upon exposition to various mycotoxins.

## 1. Introduction

In the last few decades, a growing body of evidence underlines the role of the intestinal barrier in mycotoxin toxicity. Intestinal barrier dysfunction contributes to many gastrointestinal diseases, e.g., inflammatory bowel disease or celiac disease [1]. The functioning of the intestinal barrier might be influenced by many factors including diet or lifestyle. At the molecular level, these factors may affect claudin expression and, by disturbing the connection and permeability of the epithelium, leading to many pathological conditions such as cancer development or its progression [2]. Claudins are a family of proteins that take part in the formation of tight junction connections between cells. Substances present in our everyday diet, that directly affect expression of claudins, are mycotoxins produced as secondary metabolites of various types of fungi. Mycotoxin exposure is a global health problem due to their abundance. Nevertheless, the evidence on the role of mycotoxins in carcinogenesis is still limited. This review presents a literature survey conducted to assess the role of the intestinal barrier and claudins in mycotoxin exposure. Moreover, we have evaluated the potential role of the most common mycotoxins in the regulation of the expression of claudins in gastrointestinal cancers.

## 2. Intestinal Barrier: In Health and Disease

The intestinal barrier plays a critical role in human health. First of all, it should be emphasized that the intestine is called the second brain for a significant reason. The influence of the gut on the functioning of the human body is not very well understood yet. Apart from the obvious role of participation in the absorption of water and nutrients (products of digestion), as a highly dynamic barrier between the external environment (intestinal lumen) and human tissues, it plays also a modulatory role between the gut microbiota and the central nervous system (CNS). The presence of the continuous and integral barrier allows the body to maintain homeostasis [3], and dead cells are replaced with new ones derived from intestinal stem cell niches (ISCs) [4].

The first element of the barrier is mucus. The intestinal mucus layer has a primary role in intestinal protection against mechanical, chemical, and biological attacks. Goblet cells are responsible for mucus production, and there are many types of goblet cells. The type of individual cells depends on their particular location in sections of the digestive system. Moreover, cell origin determines also the thickness of the mucosa [5,6]. Mucus consists of α-defensins, human α-defensin 5 (HD-5), and human α-defensin 6 (HD-6) (secreted by Paneth cells located in the small intestinal crypts) or secretory immunoglobulin type A (sIgA) [7,8,9,10,11,12]. The presence of these components helps to maintain the balance in the composition of the gut microbiota. Dysfunction in the production/secretion of individual proteins can lead to disturbances in the proportions of individual bacteria.

**Figure 1:**
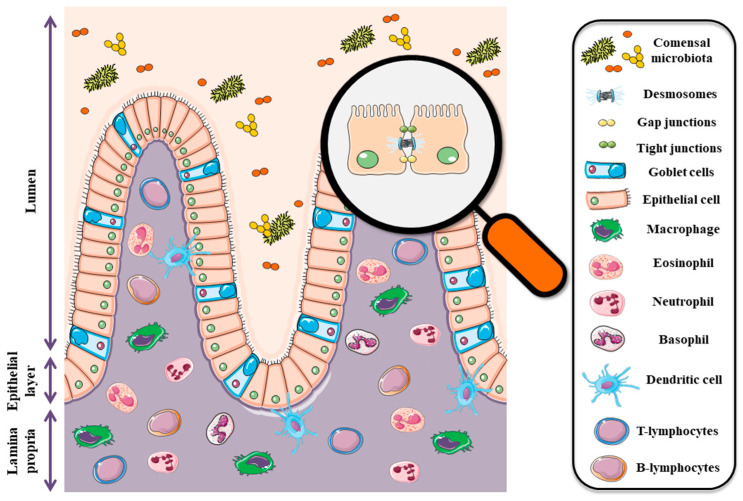
Diagram showing the intestinal barrier components. The graphical illustration was prepared by using the images from Servier Medical Art by Servier. Minor modifications were made (e.g., color of the stock images, some shapes) (https://smart.servier.com/smart_image/, accessed on 19 July 2021).

The second barrier is formed by epithelial cells and inter-epithelial tight junctions (TJ) that are primarily responsible for cellular integrity (Figure 1). These junctions are made by a number of the following proteins; claudins, occludin, junctional adhesion molecules (JAM), and tricellulin as well as cytoplasmic plaque proteins such as three zonula occludens (ZO) proteins [13]. Tight junctions regulate epithelial polarity and vectorial movement of solutes and fluids in the intercellular space [14,15,16]. Disturbances in the expression of individual proteins included in tight junctions are associated with many pathophysiological conditions, tumors in particular [17,18,19,20]. The intestinal epithelium is also the first barrier against food contaminants and is highly sensitive to *Fusarium* toxins, especially deoxynivalenol (DON) and zearalenone (ZEA). All chemical substances and biological factors, influencing the expression of genes encoding tight junction elements, affect the integrity and permeability of the digestive system barrier. Thus, in the case of mycotoxins, chronic exposure to low doses of mycotoxins leads to many pathophysiological conditions, including cancer.

The third barrier is the cells of the immune system. Intraepithelial lymphocytes (IETs) and dendritic cells (DC) deserve special attention due to their contribution to the immune response. These cells are the first line of defense against enteric pathogens [21,22]. The gut-associated lymphoid tissue (GALT), literally the largest peripheral lymphoid tissue in the body, is definitely more complex. The role of this tissue is primarily to act in the context of flora selection in order to maintain homeostasis.

## 3. Claudins in Intestines-Schedule and Function in Health

Claudins are a family of proteins responsible for the formation of tight junctions between cells. Their mass is about 20–34 kDa. The structure of the various claudins is very similar and the major components that can be distinguished are: four transmembrane domains, two extracellular loops and amino- and carboxyl-terminal tails, which are located in the cytoplasm [23]. The first extracellular loop is involved in the regulation of paracellular charge and ion selectivity due to the presence of charged amino acids. The second one is associated with interactions between adjacent claudins. The amino tail is about seven amino acids and its length and sequence is generally similar among the claudin family. On the contrary, the carboxyl-tail is much more heterogeneous with 22 to 55 amino acids. The carboxyl end contains PDZ-motif that allows claudins to interact with other TJs proteins such as zonula occludens-1 (ZO-1). Moreover, post-translational modifications (e.g., phosphorylation or SUMOylation) take place in this region that may affect the functionality of the protein [23].

The tetraspan claudin family of proteins includes 26 family members in humans. However, the occurrence of particular claudins is determined by tissue and may differ depending on the type of the organism (Table 1). Their expression and location may be modulated by many substances including hormones [2]. For example, the estrogenic-like mycotoxin- zearalenone stimulated inflammation, disrupted the intestinal microflora and decreased the expression of claudin-4 in piglet intestine [24]. It should be underlined that pathological conditions (e.g., mucosal inflammation or cancer) are mainly associated with claudins disturbances what underlines their importance [25]. Claudins can be divided into two broad categories, pore-sealing and pore-forming claudins [26,27,28]. Alternations in the expression profile of individual claudin lead to changes in the paracellular transport/absorption of ions, fluids and substances such as drugs. Individual claudins can be regulated at several steps, including; transcription, microRNA repression, trafficking and phosphorylation. Claudins classified in the “pore-sealing” group, as the name suggests, lead to the sealing of junctions, thus reducing the permeability for various solutes and compounds. This group includes claudin-1, -3, -4, -5, -7, and -19. A group with opposite properties are claudins, classified as “pore-forming” (e.g., claudin-2 and -15), which are responsible for decreasing the tightness of the epithelium and increasing the permeability for different solutes [29,30]. Pore-forming claudins are responsible for size and charge selectivity of paracellular transport via tight junctions in a large variety of epithelia [25,31]. Apart from the obvious role of creating scaffolds, the purpose of which is to mechanically support epithelial cells, tight junctions (including claudins) are involved in signal transduction within the cell. An example that clearly shows the critical role of claudins as elements of tight connections is the claudin-1 knockout mice model- loss of claudin-1 leads to severe dehydration and postnatal death in mice [32]. The presence of claudins and other tight junction elements also plays a role in the intercellular communication process, but more importantly, it maintains a balance between proliferation, differentiation, and migration [33]. As different parts of the gastrointestinal tract vary in physiological properties, and therefore also in mucosal barrier permeability, they can have different claudin expression profiles. In mice, claudin-18 expression was observed in duodenum and jejunum [34,35]. However, there are contrary statements about its occurrence in the taste tissue [35,36]. In humans, claudin-18 was observed in gastric mucosa [37]. In the esophagus, the expression of claudin-4 and -7 was observed. The expression of *CLDN1* and *CLDN5* in the stomach was restricted to the glandular epithelium in mouse tissues [35]. The differences in claudin profiles in the human tissues were shown between the fundus and antrum of the stomach [38]. Claudin-3 and -4 were reported to be expressed at the highest levels in colon and rectum in rats and human samples [38,39]. Claudin-7 in rats and mice is observed in the ileocecal region [34,39], whereas in humans it was observed in the colon and rectum [38]. Expression of -8 in mice and human intestines was found to increase along the colon toward the rectum, contrary to claudin-15, which had its expression’s peak in duodenum and jejunum [34,38,40]. Claudin-2 was predominantly expressed in the proximal intestine and maximal expression of claudin-12 was observed in mouse ileum [40] or jejunum in rat [39].

## 4. Claudins in Gastrointestinal Cancer

**Figure 2:**
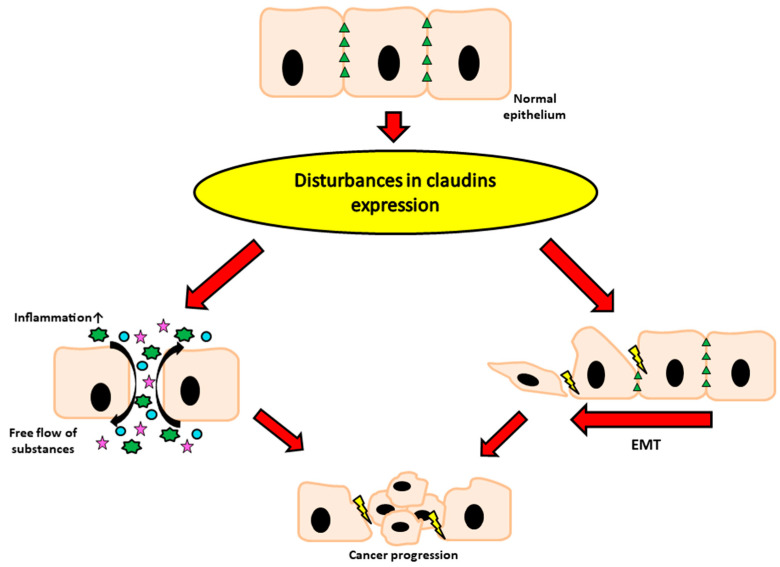
Diagram showing the consequences of abnormal claudin expression. EMT—epithelial to mesenchymal transition.

As mentioned above, it is indisputable that tight junction proteins play a huge role in maintaining physiological homeostasis. Their abnormal expression very often correlates with many types of cancers, including the reproductive and digestive system, as well as many others (Figure 2) [2,53]. Recently, TJ proteins have gained more and more attention, due to their crucial function in the pathogenesis of various diseases and the high potential for both diagnosis and treatment. As shown in Table 2, modulation of claudin expression in different cancers may vary. Moreover, their expression may be affected by many substances, including mycotoxins, which we would like to present in the next step of our article.

### 4.1. Oral Cancer

Oral cancer can attack any part of the oral cavity. It is widely believed that the most important risk factors of this type of cancer are smoking and high intake of alcohol. As oral cavity cancer has a very high mortality rate (~50%), understanding the role of claudins in this disease seems to be necessary to allow improvement of current therapeutic or diagnostic modalities [84]. Numerous studies reported that claudin-1 play a significant role in this type of cancer and its elevated expression is observed in most oral, carcinomas. Moreover, the higher the expression, the more advanced stage tumors are diagnosed, and what is worth emphasizing—it is also associated with a lower survival rate [54,55,56,57]. Patricia Pintor Dos Reis et al. showed that overexpression of *CLDN1* is associated with higher invasion of cells and aggressiveness observed in immunohistochemistry [58]. Naohisa Oku et al. explained that claudin-1 stimulates the invasiveness of oral cancer cells via activation of MT1-MMP and MMP-2 [85]. Silencing of *CLDN1* results in decreased invasive potential and proliferation of oral cancer cells [58,59]. Another very important claudin associated with the pathogenesis of oral cancer appears to be claudin-7, which has been reported to be downregulated in most cases of this type of cancer [60]. Furthermore, it was also postulated that downregulated expression of claudin-7 is associated with an increased risk of cancer recurrence and poor prognosis [61,86].

### 4.2. Esophageal Cancer

Esophageal cancer is not the most common cancer; however, the mortality rate is very high, and the prognosis is very poor, therefore this disease is considered a serious global health problem [87]. Some studies reported the involvement of TJ proteins in the character of this cancer. On the one hand, it was reported that the decreased expression of claudin-1 in tissue derived from patients was associated with poor prognosis and an increased risk of recurrence [88]. On the other hand, it was observed that increased expression of *CLDN1* results in increased proliferation and metastasis via stimulation of autophagy in human esophageal cancer cell lines. Moreover, the same team demonstrated in an in vivo model that claudin-1 is able to stimulate metastasis [62]. Elevated expression of claudin-2 was observed in the cancerous and pre-cancerous lesion [89]. Lower expression of *CLDN4* is postulated to be a risk factor and prognostic biomarker for cancer recurrence and survival rate [63,90]. Furthermore, in vitro studies have shown that claudin-4 expression regulates invasion and metastasis of cells [63]. It was also shown that Twist1 may modulate *CLDN4* expression which suggests that claudin-4 is directly associated with EMT in esophageal cancer [91]. Another claudin involved in esophageal cancer is claudin-7, which was reported to be downregulated and this disturbance may lead to cancer progression [64]. This hypothesis seems to be confirmed by later studies, which showed that claudin-7 influences the expression of E-cadherin, and thus may lead to loss of the “gate” function and stimulate EMT [42].

### 4.3. Liver Cancer

Among liver cancers, we can distinguish two major types: hepatocellular carcinoma (HCC) and bile duct cancer (cholangiocarcinoma, CC). The first one occurs more often and constitutes a serious global problem. The main risk factors are viruses, metabolic disorders, and, as was mentioned before, toxins, including aflatoxin. TJ proteins appear to be promising markers in this disease, however, we still do not know enough to use them in medical diagnosis. It was suggested that decreased expression of claudin-1 may be associated with poor survival rates and potential for metastasis and invasion [65]. At the same time, overexpression of *CLDN1* may be a positive prognostic marker after treatment [92]. However, it was also observed, that overexpression of this protein induces EMT in normal liver cells [93]. Liver metastases from primary colorectal cancer were characterized by an increased expression of claudin-1 [94]. Silencing of *CLDN1* sensitizes HepG2 to 5-fluorouracil [95]. Based on the abovementioned facts, it may be concluded that abnormal *CLDN1* expression has a very important impact on the liver. Similarly, as claudin-1, proper expression and localization of claudin-3 in cells are also very important to maintain homeostasis. Downregulation of this protein was observed in HCC; moreover, it was also found in most types of HCC cell lines. Lei Jiang et al. reported that silencing of claudin-3 in HepG2 and Huh7 cell lines stimulates changes in their morphology, increases the ability to migrate, induces foci formation in monolayer culture, and stimulates cells invasiveness [66]. All of these underline the importance of claudin-3, however, further studies are required. Claudin-7 expression is upregulated in HCC [96]. Yusuke Ono et al. postulated that claudin-4 and claudin-7 may be a useful immunohistochemical marker to distinguish HCC and CC, because the expression of these proteins is higher in these two cases than in control, and the expression of *CLDN4* and *CLDN7* is lower in HCC than in CC [97]. Earlier studies also provided evidence for this relationship [98,99]. Claudin-10 is overexpressed in most HCC patients and that expression is highly associated with poor prognosis after resection of the liver [67]. Studies conducted in in vitro models showed that abolition of *CLDN10* reduces the invasiveness of cancer cells what seems to be promising in the context of the possibility of using claudins as a target in the treatment of liver cancer [100].

### 4.4. Gastric Cancer

Gastric cancer (GC) also known as stomach cancer is one of the most common causes of human death. Each year 989,000 people are diagnosed with gastric cancer, out of which 738,000 patients die because of this disease [101]. Many factors contribute to its development, including age, tobacco smoking, diet, *Helicobacter pylori* infection, and many others [102]. Claudins are postulated to play an important role in this type of cancer as well. Claudin-1 was found to be elevated in GC. Moreover, it has been observed that increased expression of *CLDN1* gene is correlated with poor survival [68,103]. Huang J et al. showed that claudin-1 is regulated by β-catenin in gastric cancer samples [103]. Elevated expression of *CLDN1* in cancer cells results in increased proliferation, migration, invasion in gastric cancer cells, but also protects them against apoptosis [69,70]. Zhe Lin et al. reported that claudin-2 and claudin-6 are down-regulated, while claudin-11 was upregulated compared to normal tissue [104]. In contrast, Luoluo Yang et al. have not noticed any significant difference between claudin-2 expression in normal and cancerous tissue, but they found that claudin-5 expression was significantly higher and the levels of claudin-7 and claudin-8 were significantly lower than in normal tissue, both at the protein and mRNA levels [105]. Claudin-4 was observed to be overexpressed in GC, moreover, increased expression of *CLDN4* results in enhanced invasion and migration of gastric cancer cells [106]. Tsann Long Hwang et al. reported that this effect may be generated via activation of matrix metalloproteinase proteins (MMP) [71,106]. Satoshi Ohtani et al. reported that, on the one hand, decreased expression of claudin-4 was associated with lower tumor aggressiveness, but on the other hand, low expression of *CLDN4* was associated with poor prognosis and survival [72]. Studies conducted on the GC cell line demonstrated that silencing of *CLDN4* expression leads to increase resistance to chemotherapy [73]. Moreover, the abolition of claudin-4 stimulates migration, invasion, and proliferation [73]. Abnormal expression of *CLDN6* was also observed in tissue obtained from patients with gastric cancer. Furthermore, up-regulated expression of claudin-6 is connected with poor prognosis and survival [74,107]. Overexpression of claudin-6 both in gastric cancer cells and in vivo models results in increased migration, proliferation, and invasiveness [74,75]. Interestingly, claudin-6 may stimulate invasion and migration via claudin-1 and thus activate MMP proteins [108]. Site Yu et al. proposed that claudin-6 may stimulate EMT by affecting YAP1-SNAIL1 axis [74]. Overexpression of Claudin-7 correlates with poor prognosis and a high possibility of lymph nodes metastasis [18,76]. Studies conducted on cells and animal models showed that up-regulated claudin-7 expression has a great impact on cell proliferation, migration, invasion, and colony formation [75,76]. Claudin-11 was found to be down-regulated in GC tissue, furthermore, silencing of *CLDN11* in gastric cancer cell line was responsible for increased motility and invasiveness [77]. Kyong Hwa Jun et al. presented that negative regulation of claudin-11 may be useful as a marker of poor prognosis in patients with gastric cancer [18]. It seems that this thesis was confirmed by later research [109,110,111]. Claudin-10 and claudin-17 were down-regulated in GC tissues, claudin-14 was observed to be up-regulated [45].

### 4.5. Colon Cancer

Colorectal cancer (CRC) is one of the most common cancers worldwide. In the UK, 11% of new cancer cases are CRC [112]. Nevertheless, it is estimated that the incidence of colorectal cancer is still decreasing [113]. It may be associated with increased knowledge about nutrition. Claudins are undoubtedly very important in the formation, progression, and metastases of this cancer. Moreover, CRC appears to be the best-known neoplasm in terms of claudins. Already in 2005, Punita Ghawan et al. reported that claudin-1 has a significant role in colon cancer. They showed that the expression of *CLDN1* is elevated in commercially available cell lines (HT29, SW480, and SW620, but not in HCT116) and in samples obtained from patients; furthermore they demonstrated that localization of claudin-1 is different than in normal tissue. In vivo results reported by the same team, showed that abolishment of the expression of *CLDN1* stimulates liver metastasis and tumor size, at the same time providing that one of the mechanisms in the regulation of claudin-1 expression may be the regulation of E-cadherin and β-catenin/Tcf signaling pathway [78]. These results seem to be consistent with the other reports [114,115]. Amar B Singh et al. proposed another mechanism of action and showed that cldn1 may regulate E-cadherin via ZEB-1 (Zinc Finger E-box binding homeobox-box1) and thus increasing the invasiveness of cells [79]. In turn, Jillian L Pope with colleagues presented that claudin-1 is closely associated with the Notch-signaling pathway, and thus affects the behavior of cells [116]. Other authors showed that pyruvate kinase M2 (PKM2) increased the expression of cldn1 in Caco-2 and SW480 cell lines via the epidermal growth factor receptor (EGFR)- protein kinase C (PKC) pathway [117]. All these studies have clearly demonstrated how complex the mechanism of action of claudin-1 is. Recent results shed new light on the use of *CLDN1* in the diagnostics of CRC. It was indicated that *CLDN1* may be useful in the future as an imaging agent in fluorescence-guided surgery [118]. Interestingly, the use of claudin-1 during confocal endomicroscopy seems to be promising, as it can significantly increase the detection of the precancerous lesion within the intestine based on the expression of this protein—as it was shown in an animal model [119]. In contrary to previously described results, Murray B Resnick et al. reported that down-regulated expression of claudin-1 in tissue obtained from patients in II stage colon carcinoma was associated with poor prognosis and a high possibility of tumor recurrence [120]. All of this underlines the importance of proper expression of claudin-1 in our organism because both too high and too low expression may lead to homeostatic imbalance. Claudin-2 was also reported to have an important role in colon cancer progression. It was observed that its expression is higher in CRC than in normal, physiological tissue [80]. It was also suggested that *CLDN2* may act via EGFR transactivation and that forced claudin-2 expression leads to increased proliferation of cells and significantly greater tumor growth in vivo [80]. In turn, claudin-3 was observed to be decreased in CRC. Studies conducted on cells with silencing *CLDN3* expression showed that with the abolition of claudin-3 expression, an increase in cell invasion and migration occurs [81]. Similar to claudin-3, claudin-7 is also down-regulated in colon cancer tissues [82]. Bhat et al. demonstrated that claudin-7 may have anti-cancer properties because its forced expression in in vitro models led to reduced invasiveness, colonies formation, proliferation, and ability to grow, while abolition resulted in enhancement of these properties. In in vivo models decreased expression of claudin-7 led to the lower weight of the tumor [82]. Similar results, but on a different cell line have been shown recently, which seems to confirm previous studies [83]. Moreover, it was noticed that *CLDN7* expression correlates with the potential for metastasis in CRC- it has been proposed that decreased expression of claudin-7 may be a predictor of liver metastasis [121].

## 5. Environment Contamination by Mycotoxins and Their Occurrence in Food and Feed

Mycotoxins are chemical compounds considered as secondary metabolites of fungi, generally from *Fusarium*, *Aspergillus*, and *Penicillium* genus. Worldwide, mycotoxins have significant implications for human and animal health, as well as for the economy and international trade [122]. Mold that can produce mycotoxins grows on numerous foodstuffs such as cereals, dried fruits, nuts, and spices. Their growth might occur either before harvesting or after harvesting, during storage, on/in the food itself often under warm, damp, and humid conditions. It is worth emphasizing that most mycotoxins are chemically stable even during food processing, and that their neutralization is only available for feed [123]. The commonness of occurrence of fungi and their spreading is mainly due to the climate conditions that favor the growth and multiplication of fungi, food spoilage and then favor mycotoxin production. These conditions are very important in the harvesting, transport, and storage—the processes that depend on good practices and habits of people responsible for them [124]. Several hundred different mycotoxins are known, but the most commonly observed mycotoxins that present a concern to human health and livestock include aflatoxin B1 (AFB1), ochratoxin A (OTA), patulin (PAT), fumonisins (FBs), zearalenone (ZEA), nivalenol (NIV) and deoxynivalenol (DON) [125]. The presence of individual mycotoxins is also partially determined by the type of crops e.g., in the case of maize, widespread occurrence of fumonisins and deoxynivalenol is observed. This review aims to collect and analyze the information about the effects of individual mycotoxins on the gastrointestinal tract in the context of claudins as a core element of tight junctions.

## 6. Mycotoxins and Human Health Especially the Health of the Gut and the Entire Digestive Tract

It is generally known that mycotoxins possess harmful effects both on human and animal well-being. The influence on human health is mainly observed in the area of the reproductive system (hyper estrogenic syndrome, precocious puberty), hepatotoxicity (especially hepatocellular carcinoma) immunotoxicity, or genotoxicity (carcinogenic, mutagenic, teratogenic), and nephrotoxicity (nephropathies and urinary tract tumors) [126,127,128,129,130]. Interestingly, it was also reported that some toxins may stimulate neurodevelopmental toxicity. In vivo study showed that ochratoxin A affects differentiation of almost all neural cells [131]. Moreover, exposure to OTA was also reported to be connected with pathobiology of autism in autistic children what underlines the importance of controlling the presence of mycotoxins in our diet [132,133]. In zebrafish model, authors observed that aflatoxin B1 may influence neurobehavior and neurodevelopment [134,135]. Similar results were obtained in response to exposure to zearalenone [136].

The negative effect of mycotoxins is mainly observed in the case of long-time exposure to low doses of mycotoxins. It is also worth emphasizing that the gastrointestinal tract is the first element that is directly exposed to the toxic effects of mycotoxins. The limitation in exploring the impact of mycotoxins on the human body is primarily the quantification of lifetime individual exposure. Analysis of 74,821 samples of (feed and feed raw materials) from 100 different countries revealed the presence of at least one of the mycotoxins (64% of samples was co-contaminated with at least two mycotoxins) what seems to be important when we know that people may be exposed to these secondary metabolites after consuming food products derived from animals exposed to high levels of mycotoxins in their feed [124,137]. The analyzed data present the global scale of the problem and provoke reflection because of potential health risks. The agency responsible for the preparation of the guidelines is the European Food Safety Authority (EFSA). To protect people against adverse effects of mycotoxins, EFSA established a total tolerable daily intake for humans (Table 3).

The unavoidable presence and chronic human exposure to mycotoxins lead to the occurrence of many pathological conditions, ranging from disturbances in the composition of the gut microbiota to neoplasms, e.g., of the liver [141,142]. The role of mycotoxins in modulating the immune response is also not negligible. GALT, as the tissue responsible for regulating many immune processes, plays a crucial role. Its proper functioning allows maintaining the balance between many organs, which is why the intestine is called the second brain for a reason. Therefore, the next step in our review was to compile the information about the effects of the mycotoxins on a healthy gastrointestinal tract with emphasis on modulation of tight junction proteins if it was reported.

### 6.1. Aflatoxins

Aflatoxins (AF) are secondary metabolites of fungi produced by *Aspergillus* molds (*Aspergillus flavus*, *Aspergillus parasiticus*, *Aspergillus nomius*). In 1987, aflatoxins (including aflatoxin B1, B2, G1, G2, and M1) were classified as Group 1 on the basis of the International Agency for Research on Cancer (IARC) evaluation [143]. Group 1 classifies chemical compounds as clearly influencing the neoplastic process in humans. Aflatoxin B1 (AFB1) is considered one of the most harmful compounds produced by *Aspergillus*. Aflatoxin contamination is mainly reported in maize, peanuts (and generally ground nuts) and their products, oilseeds, and pulp [144]. Aflatoxin B1 is rapidly absorbed into the blood from the gastrointestinal tract (GIT). In the bloodstream, aflatoxin B1 is metabolized into its toxic metabolite AFB1-8,9-exo-epoxide (AFBO) [145]. AFs have carcinogenic, hepatotoxic, teratogenic, mutagenic, and immunosuppressive effects, with the liver as the organ responsible for detoxification is most affected by the negative effects of aflatoxins [146]. In vivo studies in pigs show a clear effect of AFB1 on the functioning of the digestive system, in particular the jejunum. Increased serum diamine oxidase (DAO) activity was observed in AFB1 treated group, indicating that AFB1 supplementation damages intestinal barrier integrity. Moreover, pigs fed with the AFB1 diet exhibited significantly decreased mRNA abundance of ZO-1 in jejunal mucosa, which supports the thesis made above, that aflatoxin B1 has a destructive effect on gut barrier integrity [147]. Reduced final body weight (BW) in pigs (AFB1 diet) may also be associated with a decrease in mRNA expression of sodium-glucose co-transporter 1 (SGLT1) and solute carrier family 7 member 1 (SLC7A1) in jejunal mucosa [147,148]. Both SGLT1 and SLC7A1 act as intestinal epithelium transporters of nutrients from the intestinal lumen (glucose and cationic amino acids respectively) [149,150,151]. In mice exposed to aflatoxin B1 and aflatoxin M1 separately and in combination, it was observed that villus height was reduced, while the crypth depth was deepened compared to non-treated mice. Moreover, the expression of claudin-1 and ZO-1 were significantly decreased [152]. Disturbed barrier function via affection claudin-1 expression was also observed in broiler chicks, but in this study, aflatoxin B1 stimulated the expression of *CLDN1* [153].

### 6.2. Fumonisins

Fumonisins are secondary metabolites produced by a number of *Fusarium* species, especially *Fusarium verticillioides* and *Fusarium proliferatum* [154]. Three main chemical compounds belong to the Fumonisis group; namely FB1, FB2, and FB3 [155]. Maize and its derivatives (corn-based food) are one of the main sources of fumonisins, which can potentially influence the occurrence of pathological conditions in both livestock and humans. Fumonisin B1 is the most prevalent member of the Fumosin family. According to the IARC opinion, fumonisin B1 was classified as possibly carcinogenic to humans. The last update of the data took place in 2002, and the decision was made based on the results of in vivo studies in mice and rats (at present, the extensive scope of publications clearly indicates the association of fumonisins and the cancer process in the human body). Currently, the vast majority of the data is focused on the association between fumonisin B1 and esophagus cancer (OC) [156,157,158,159]. According to available evidence, the molecular mechanism of action is linked to sphingolipid metabolism [160,161]. Due to the similarity to two critically important molecules (sphinganine and sphingosine), the ceramide synthesis pathway is blocked. The consequence of the inhibition process is the inhibition of the sphingolipid biosynthetic pathway (disrupted sphingolipid metabolism) [162,163]. In IPEC-J2 cells, FB1 leads to decreased viability, decreased expression of tight junction proteins (*CLDN1*, *OCLN*, and *ZO-1*), and altered expression of mucin genes (*MUC1*, *MUC2*). Moreover, the permeability of IPEC-J2 monolayer was also disturbed after exposition to fumosin B1 [164]. It was also observed that FB1 decreased transepithelial electrical resistance (TEER) value in IPEC-J2 cells [165]. Similar results were obtained on pig iliac endothelial cells (PIECs) [166]. Exposure to fumonisin was also postulated to be responsible for the decrease in the diversity of the bacterial flora [167].

### 6.3. Zearalenone

Zearalenone (ZEA) is a non-steroidal estrogenic mycotoxin. The species of fungi that are responsible for the biosynthesis and secretion of the ZEA mycotoxin are mainly *Fusarium graminearum* (*Gibberella zeae*), *Fusarium culmorum, Fusarium crook-wellense*, *Fusarium semitectum* and *Fusarium equiseti* [146]. The abovementioned fungi species are responsible for the contamination of most cereal crops worldwide. According to the IARC classification, ZEA is not classifiable as to its carcinogenicity to humans. It owes its estrogenic properties to the presence of a macrocyclic lactone ring with a spatial arrangement similar to that of steroid hormones (structurally similar to 17β-estradiol) [168,169,170]; hence the destructive effect of zearalenone on the reproductive system. Until now, 5 metabolites of the ZEA have been described, i.e., α-Zearalenol (α-ZEA), α-Zearalanol (α-ZAL), Zearalanone (ZON), β-Zearalenol (β-ZEA), β-Zearalanol (β-ZAL), with individual metabolites possessing different estrogenic properties [171]. Due to the presence of the lactone ring, ZEA is known to be heat stable up to 150 °C and does not degrade during food and feed processing; this presents some sort of challenge in terms of removing mycotoxins, particularly from food chain products [172]. The following events related to the presence of zearalenone in the human body are distinguished: altered progesterone level, hyper-estrogenic syndrome, precocious puberty, decreased sperm count, decreased serum testosterone level, and infertility [126,127,173,174,175]. Additionally, disruption of blood coagulation and changes in haematological parameters in rats were found [176,177]. Unfortunately, the context of the intestinal barrier is still little recognized. In the intestine of Juvenile Grass Carp (*Ctenopharyngodon idella*), ZEA affected intestinal integrity via affecting tight junctions [178]. In the intestine of piglets under the influence of ZEA, the expression of claudin-4 was reduced and the intestinal microbiota was disturbed [24]. Zearalenone was also postulated to decrease claudin-4, occludin, and connexin-43 in tissue derived from rats [179,180]. Similar to FB1, ZEA is able to affect mucin genes [180]. In addition, ZEA was reported to alter the morphological structure of the villi in the jejunum [179].

### 6.4. Deoxynivalenol

Deoxynivalenol, also known as vomitoxin, is the most common member of the trichothecenes group which include also nivalenol. *Fusarium*, *Myrothecium*, *Phomopsis*, *Stachybotrys*, *Trichoderma*, *Trichothecium*, and other species are responsible for their production [181,182]. DON frequently contaminates cereal grains such as maize, wheat, oats, barley, and rice. Among trichothecenes group, type A and type B are the most concerning due to their broad and highly toxic nature, with DON being classified as trichothecenes B. In the IARC assessment, DON was classified in Group 3 so it is not classifiable as to its carcinogenicity to humans. DON possesses the following effects: cytotoxicity, steroidogenesis disruption, affected mRNA expression of genes responsible for regulating the integrity and permeability of the intestinal barrier, increased reactive oxygen species (ROS) production, inhibition of cellular protein synthesis, and ribosomal stress syndrome [183,184,185,186,187]. Changes in the functioning of the intestinal barrier were observed both in in vitro and in vivo studies. It was observed that DON decreases TEER value in IPEC-2 cells [188,189]. The expression of occludin, claudin-3, and claudin-4 is also modulated by DON which was showed both in in vitro and in vivo models [188,190,191,192]. In human non-cancerous intestinal epithelial cell line (HIEC-6), decreased expression of claudin-1 and increased expression of inflammatory-related interleukins was observed [193]. Moreover, it was also found that DON may affect another very important compound associated with an intestinal barrier—trefoil factors (TFFs), which are involved in repairing and protecting intestinal mucus. Shuai Wang et al. showed that DON decreases expression of TFF2 and TFF3, which may suggest that it is another pathway altered by deoxynivalenol in the intestine [190]. Induction of immune response was also observed [24,191]. It was postulated that the pathway connected with an intestinal disturbance caused by DON may be MAPK p44/42 pathway [189,192,194]. In the context of changes in the intestinal biota, an increase in abundance of the *Lactobacillus* genus was observed (DON and ZEA treated group), suggesting that members of this genus could play a key role in the detoxification of dietary DON and ZEA in pigs [195]. Basolateral DON exposition caused inhibited intestinal stem cell activity through the Wnt/β-catenin pathway [196]. Based on this study, Hikaru Hanyu et al. decided to go one step further, demonstrating that basolateral exposure was more toxic than luminal DON exposure in terms of intestinal barrier functions and stem cells [197]. It is worth mentioning that DON toxicity observed in vitro and in vivo might be different, possibly due to biological barrier function. It was observed that in a zebrafish model, DON does not cause any toxic effect, as suggested by in vitro results. Shu Guan et al. demonstrated that DON is transformed via gut microbes to the depoxyated form of DON (DOM-1) which is less toxic than DON itself [198]. A similar effect was observed in zebrafish larvae exposed to DON microinjections and aqueous solutions, where no effect was observed, which in consequence suggests that DON is not capable to pass through biological barriers [199]. This fact underlines the importance of the intestinal barrier in response to exposition to mycotoxins.

### 6.5. Patulin

Patulin (4-hydroxy-4H-furo [3,2c]pyran-2(6H)-one, PAT), water-soluble polyketide lactone, is a secondary metabolite of filamentous fungi (toxigenic molds) such as *Penicillium*, *Aspergillus*, and *Byssochlamys* species. PAT was firstly isolated as a compound with antimicrobial activity in the 1940s from *Penicillium patulum*. Among all fungi listed above, *Penicillium expansum* is the main source of patulin contamination in apples, pears, and their derived products, and it also causes decay in fruits [200]. Over time, detrimental effects of patulin on animal organisms were discovered. Patulin has been classified by IARC in group-3 (not classifiable as to carcinogenicity to humans) [201,202]. The potential effect of patulin on the gastrointestinal tract results primarily from the reduction of TJs’ mRNA (including ZO-1 and Occludin) and degeneration of intestinal villi [203,204]. In addition, in animal models, PAT exposure has been shown to lead to epithelial degeneration, hemorrhage, ulceration of gastric mucosa, reduction in the number of goblet cells in villi and crypts [205,206]. From the molecular biology point of view, change in the composition of tight junctions or degradation of epithelial cells leads to impaired permeability (TEER increase), which results in a loss of balance between the external environment and the organism [207,208]. Moreover, changes in the claudin distribution patterns are observed (for claudins 1, 3, and 4, the staining pattern became quite diffuse compared to the controls, large gaps were also observed to have appeared in the ‘chicken wire’ pattern) [209]. Nevertheless, the effect of patulin on non-cancerous junctional cells has not been evaluated yet.

**Figure 3:**
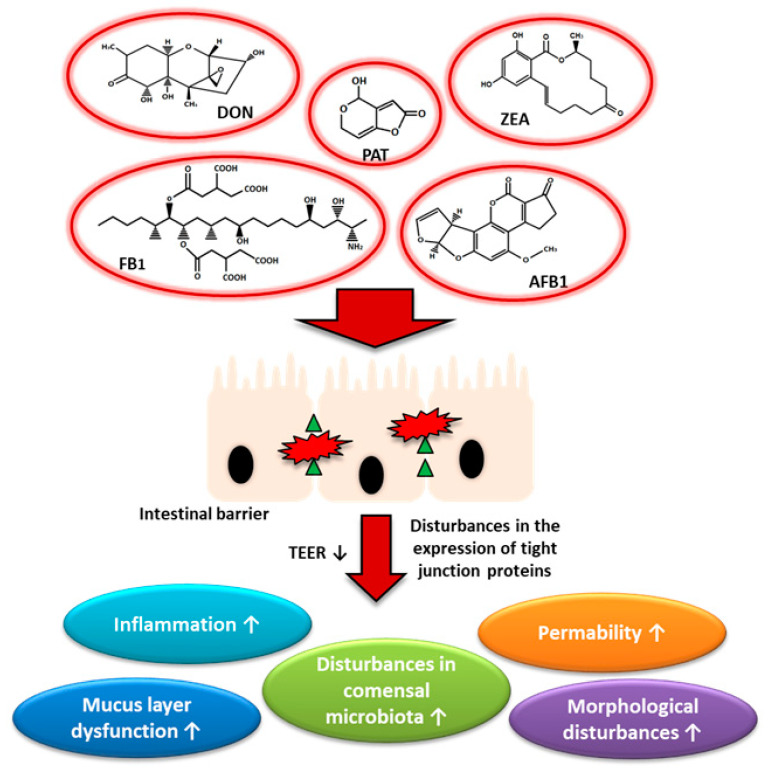
Mycotoxins and their influence on intestinal physiology. DON—deoxynivalenol, PAT—patulin, ZEA—Zearalenone, FB_1_—Fumosin B1, AFB1—Aflatoxin B1.

Summarizing all the collected information, it can be concluded that the range of mycotoxins activity in the area of cells of the human body is very wide. A critical role in the penetration of mycotoxins into the body is played by the gastrointestinal barrier, which is the first protective element. Individual mycotoxins differently influence the integrity of the gastrointestinal barriers. Some of them may affect the expression of tight junction proteins and thus disturb homeostasis in the gut (Figure 3). Interestingly, a growing body of evidence shows that naturally co-occurring mycotoxins may have a greater impact on the intestinal barrier. For example, mixed doses of *Fusarium* toxins are more harmful and stimulate an immune response more than individual mycotoxins [210]. Many of the areas, such as the influence of mycotoxins on the nervous system of the digestive tract or the immune system, require better understanding. Furthermore, it seems necessary to investigate the effect of mixed doses of mycotoxins given that the presence of more than one toxin in food is very common. Expanding the scope of our knowledge about mycotoxins has the potential to provide a better understanding and the possibility to eliminate the side effects of mycotoxins both on human and animal health.

## 7. Mycotoxins and Their Association with Claudins in Gastrointestinal Cancers

Over the past two decades, we can observe a growing body of evidence that highlights the importance of various mycotoxins in the everyday diet in different parts of the digestive system. For example, it was found that Aflatoxin B1 is one of the risk factors associated with hepatocellular carcinoma [142]. Nevertheless, there is still little known about the relationship between mycotoxins and claudins. Hereby, we described the effects of various mycotoxins on claudin expression in colorectal cancer cell lines as the most popular model in research focusing on cytotoxicity of mycotoxins and the barrier function. A lot of studies focused on molecular aspects and the effects of various mycotoxins on colon cancer cells in vitro. It is generally known that mycotoxins are able to reduce the viability of various cells, not only cancerous. Thus, it is important to understand their mechanism of action, but it is very difficult due to their variety. One of the mycotoxin mentioned in this review is deoxynivalenol. It was verified on HT-29 cell line that DON inhibits proliferation, stimulates DNA damage, increases expression of p53, leads to release of cytochrome c from the mitochondria, stimulates changes in *Bcl-2*, *Bax*, and *Bid* expression and then as a consequence induces caspase-dependent apoptosis. Moreover, deoxynivalenol in a study conducted on Caco-2 and T84 cell lines significantly decreased monolayer integrity in TEER assay [211]. This implies that DON leads to changes in tight junction, possibly by modulating the expression of various tight junction proteins. As an example, we can cite studies conducted by other researchers, where they showed that DON alters the expression of claudin-4 in Caco-2 cell line [188,212]. Other mycotoxins have also been investigated for modulation of tight junction protein expression. Alejandro Romero et al. reported that aflatoxin B1, fumonisin B1, ochratoxin A and T-2 toxin (T2) significantly reduced monolayer integrity and decreased the expression of claudin-3, claudin-4, and occludin in Caco-2 cell line [213]. Patulin leads to a reduction in transepithelial electrical resistance values, however, no changes in claudin expression were observed [204]. It is worth noticing that another study showed that although the claudin’s expression was not disturbed by patulin, their localization has changed [209,214]. The combination of mycotoxins may either affect the intestinal barrier. ChenQing Wu et al. showed that the mixture of aflatoxin M1 (AFM1), ZEA, and OTA affects the morphology of TJ proteins and thus disturbs intestinal permeability [215]. It was also observed that a combination of AFM1 and OTA presents a synergistic effect together and that they may damage the intestinal barrier [216]. However, the expression of claudins in this study has not been investigated. Gao et al. presented that the mixture of AFM1 and OTA disturbs the expression of claudin-3 and claudin-4 in Caco-2 cells and that these toxins present a synergistic effect [217]. It has been postulated that the combination of AFM1 and AFB1 may also influence the intestinal barrier function [152]. Nevertheless, mycotoxins can act as a double-way axis. It was observed that zearalenone is able to stimulate the proliferation of colon cancer cells (HCT116), but what is worth emphasizing- it all depends on the concentration, because only low doses stimulate proliferation and migration, while at high doses ZEA is cytotoxic to these cells [218]. Similar results, including ZEA derivatives, were observed in MCF-7 cells and in prostate cancer cells [219,220]. It seems to be very worrying, not only for healthy people but especially for patients with cancer, who are exposed to low doses of this mycotoxin in their everyday diet. Nevertheless, it is hard to compare in vitro results with in vivo, because the absorption of toxins in cells culture may be different. Further studies would be necessary to discover the role of ZEA and its derivatives and other mycotoxins which are poorly understood in colon cancer cell lines including the role of tight junction and epithelial permeability. At the same time, it underlines the significance of the detection of toxins in our food and the need to understand the exact mechanisms of action of these toxins in order to consider them in the context of anticancer properties in general. All the information discussed above has been summarized in the Table 4.

## 8. Conclusions

It is generally known that the intestinal barrier plays a crucial role in the functioning of the organism. Its disturbance may be associated with many pathological conditions. Some compounds, such as mycotoxin, may induce changes in their structure and thus lead to numerous disorders. As mycotoxins are known to be harmful to human and animal health, there is still little known about their effect on claudin expression in a healthy gut. It is also worth highlighting that active metabolites of main mycotoxins may be more toxic and cause even more detrimental biological effect in cells than a mycotoxin itself. For example, α-ZOL is reported to have even more estrogenic effect than ZEA itself. Thus, it should be also taken into account that not only mycotoxins but also their metabolites might participate in intestinal barrier dysfunction. In recent years, claudins gain more and more attention due to their diagnostic and therapeutic potential. Understanding the connection between mycotoxins and claudins may shed new light on both treatment options of gastrointestinal cancers and protection against adverse effects caused by mycotoxins present in our everyday diet.

## Figures and Tables

**Table 1:** Claudins expression in various parts of the gastrointestinal tract (GI) in mammals. * Distinguished parts of the colon.

Part of GI Tract	Claudins				References
	Human	Mouse	Rat	Pig	
Mouth	1, 4, 7, 8, 17	1, 2, 3, 4, 6, 7, 10, 11, 12, 17, 18, 23	-	4, 7	[35,36,41]
Esophagus	2, 3, 4, 7, 8, 12, 15, 18	-	-	-	[38,42,43]
Stomach	10, 11, 14, 17, 18, 23	1, 3, 5, 6, 11, 18	-	1	[35,37,38,44,45,46]
Duodenum	1, 2, 3, 4, 7, 8, 12, 15, 18	1, 2, 3, 4, 5, 7, 8, 9, 10, 11, 12, 14, 15, 18	1, 2, 3, 4, 5, 7, 8, 12	1, 3, 4, 5	[34,38,39,40,47,48,49]
Jejunum	2	1, 2, 3, 4, 5, 7, 8, 9, 10, 11, 12, 13, 14, 15, 18	1, 2, 3, 5, 7, 12	1, 3, 4, 5	[34,38,39,40,46,47,48,49,50,51]
Ileum	2, 3, 4, 7, 8, 12, 15, 18	1, 2, 3, 4, 5, 7, 8, 9, 10, 11, 12, 13, 14, 15	1, 2, 3, 5, 7, 8, 12	1, 3, 4, 5	[34,38,39,40,46,47,48,49]
Cecum	-	1, 2, 3, 4, 5, 7, 8, 9, 10, 11, 12, 14, 15	-	-	[34]
Colon	-	1, 2, 3, 4, 5, 7, 8, 9, 10, 11, 12, 14, 15	1, 2, 3, 4, 5, 7, 8, 9, 12	1, 4	[34,39,40,46,52]
*Ascending* *	2, 3, 4, 7, 8, 12, 15, 18	-	-	-	[38]
*Transverse* *	2, 3, 4, 7, 8, 12, 15, 18	-	-	-	[38]
*Descending* *	2, 3, 4, 7, 8, 12, 15, 18	-	-	-	[38]
*Sigmoid* *	2, 3, 4, 7, 8, 12, 15, 18	-	-	-	[38]
rectum	1, 2, 3, 4, 7, 8, 12, 15, 18	-	3	-	[26,38,42]

**Table 2:** Summarized information about claudin proteins expression in GI cancer cell line. ↑—up-regulated, ↓—downregulated.

Cancer	Claudin	Expression	Described Effects on Cells	References
Oral	1	↑	Invasiveness ↑ Proliferation ↑	[54,55,56,57,58,59]
	7	↓	Invasiveness ↑	[60,61]
Oesophageal	1	↑	Proliferation ↑ Metastasis ↑ Invasiveness ↑	[62]
	4	↓	Growth ↑ Colony formation ↑ Invasiveness ↑	[63]
	7	↓	Invasiveness ↑ Metastasis ↑ Tumour progression ↑	[64]
Liver	1	↓	Invasiveness ↑ Metastasis ↑	[65]
	3	↓	Invasiveness ↑ Metastasis ↑ Colony formation ↑	[66]
	10	↑	Angiogenesis ↑ Invasiveness ↑	[67]
Gastric	1	↑	Apoptosis ↑ Invasiveness ↑ Migration ↑ Colony formation ↑	[68,69,70]
	4	↑	Invasiveness ↑ Migration ↑	[71,72]
	4	↓	Migration ↑ Proliferation ↑ Invasiveness ↑	[73]
	6	↑	Migration ↑ Proliferation ↑ Invasiveness ↑ Colony formation ↑	[74,75]
	7	↑	Migration ↑ Proliferation ↑ Invasiveness ↑ Colony formation ↑ EMT ↑	[75,76]
	9	↑	Migration ↑ Proliferation ↑ Invasiveness ↑	[75]
	11	↓	Migration ↑ Invasiveness ↑	[77]
Colorectal	1	↑	Growth ↑ Colony formation ↑ Migration ↑ Invasiveness ↑	[78,79]
	2	↑	Colony formation ↑ Proliferation ↑	[80]
	3	↓	Proliferation ↑ Invasiveness ↑ EMT ↑	[81]
	7	↓	EMT ↑ Colony formation ↑ Growth ↑ Invasiveness ↑	[82,83]

**Table 3:** Total tolerable daily intake present for mycotoxins described in this review based on EFSA reports.

Mycotoxin	Total Tolerable Daily Intake	References
Aflatoxins	Not established	-
Fumonisin B1	1 µg/kg	[138]
Zearalenone	0.25 µg/kg	[139]
Deoxynivalenol	1 µg/kg	[140]
Patulin	Not established	-

**Table 4:** Summarized information about mycotoxins and their relationship to claudins in colon cancer cell lines. * only markedly affected, but distribution was disturbed, ↓ lower value/expression, AFM1—aflatoxin M1, ZEA—zearalenon, OTA—ochratoxin A, AFB1—aflatoxin B1.

Mycotoxin	Cell Line	TEER Values	Targeted Claudin	References
Aflatoxin B1	Caco-2	↓	*CLDN3* ↓	[213]
Ochratoxin A	Caco-2, HT-29-DR	↓	*CLDN3*, *CLDN4* ↓	[213,221,222]
Patulin	Caco-2	↓	*CLDN1*, *CLDN3*, *CLDN4*) ↓ *	[204,209,213]
T-2 toxin	Caco-2	↓	*CLDN3*, *CLDN4* ↓	[213]
Fumonisin B1	Caco-2	↓	*CLDN3*, *CLDN4* ↓	[213]
Deoxynivalenol	Caco-2, T84, HT-29-DR	↓	*CLDN4* ↓	[188,212]
AFM1 + ZEA + OTA (combination)	Caco-2	↓	*CLDN3*, *CLDN4*	[215]
AFM1 + OTA	Caco-2	↓	*CLDN3*, *CLDN4*	[217]
AFM1 + AFB1	Caco-2	↓	*CLDN1*	[153]

## Data Availability

Not applicable.
